# Selenium Utilization Strategy by Microalgae

**DOI:** 10.3390/molecules14124880

**Published:** 2009-11-30

**Authors:** Hiroya Araie, Yoshihiro Shiraiwa

**Affiliations:** Graduate School of Life and Environmental Sciences, University of Tsukuba, 1-1-1 Tennoudai, Tsukuba, 305-8572, Japan

**Keywords:** *Emiliania huxleyi*, selenoprotein, selenite uptake, alga, thioredoxin reductase

## Abstract

The diversity of selenoproteins raises the question of why so many life forms require selenium. Selenoproteins are found in bacteria, archaea, and many eukaryotes. In photosynthetic microorganisms, the essential requirement for selenium has been reported in 33 species belonging to six phyla, although its biochemical significance is still unclear. According to genome databases, 20 species are defined as selenoprotein-producing organisms, including five photosynthetic organisms. In a marine coccolithophorid, *Emiliania huxleyi* (Haptophyta), we recently found unique characteristics of selenium utilization and novel selenoproteins using ^75^Se-tracer experiments. In *E. huxleyi*, selenite, not selenate, is the main substrate used and its uptake is driven by an ATP-dependent high-affinity, active transport system. Selenite is immediately metabolized to low-molecular mass compounds and partly converted to at least six selenoproteins, named EhSEP1–6. The most (EhSEP2) and second-most abundant selenoproteins (EhSEP1) are disulfide isomerase (PDI) homologous protein and thioredoxin reductase (TR) 1, respectively. Involvement of selenium in PDI is unique in this organism, while TR1 is also found in other organisms. In this review, we summarize physiological, biochemical, and molecular aspects of selenium utilization by microalgae and discuss their strategy of selenium utilization.

## 1. Se Requirement for Growth of Microalgae

*Emiliania huxleyi*, an oceanic, unicellular haptophycean calcifying alga, is an abundant coccolithophorid known to fix a large amount of carbon and produce a huge biomass during blooms. Great attention has been paid to this organism because its influence on the global carbon cycle has a large impact on the environment. This alga uses two kinds of carbon fixation reactions, namely photosynthesis and calcification, for the production of organic metabolites and unique CaCO_3_ crystals that cover cells, respectively. *E. huxleyi* requires nanomolar levels of selenium (Se) for its growth. The selenite ion [Se(IV)] is the dominant molecular species used by this alga, and the optimum concentration is 1 nM, but selenite is toxic at concentrations greater than 1 μM [1]. The selenate ion [Se(VI)] is also effective for maintaining growth, but concentrations greater than 1 µM are required. As the concentrations of selenite and selenate in the *Emiliania* growth habitat in the ocean are 0.1–0.2 and 0.1–1.0 nM, respectively [2], only the selenite ion is useful for *Emiliania* growth.

Studies have reported that some algae require Se for growth. When Se was added to the culture medium, growth was stimulated in the diatom *Thalassiosira pseudonana* [3], *Chysochromulina breviturrita* in Haptophyceae [4], the dinoflagellates *Gymnodinium catenatum* and *Alexandrium minutum* [5,6], and other algae [7,8,9,10,11,12,13,14,15,16] (Table 1). Such a growth-stimulating effect is greater for selenite than selenate ions [17]. In seawater, inorganic Se is present as selenate (SeO_4_^2-^) and selenite (SeO_3_^2-^) at a ratio of about three to one in the euphotic zone [2], while in soils almost all Se is present in the form of selenate, and selenite is scarce. However, studies on the effect of selenite in the unicellular green alga *Chlamydomonas reinhardtii,* a model algal organism, showed only a little stimulative effect on growth [30].

**Table 1:** Phytoplankton species that were demonstrated to require selenium for their growth.^ a^

Phylum	Species	Reference
**Diatoms**	*Amphiprora hyalina*	[7]
	*Chaetoceros debilis*	[7]
	*Chaetoceros pelagicus*	[7]
	*Chaetoceros vixvisibilis*	[7]
	*Coscinodiscus asteromphalus*	[7]
	*Corethron criophilum*	[7]
	*Ditylum brightwellii*	[7]
	*Skeletonema costatum* (strain 18c NEPCC)	[7]
	*Skeletonema costatum* (strain 611 NEPCC)	[7]
	*Skeletonema costatum* (strain 616 NEPCC)	[7]
	*Stephanopyxis palmeriana*	[7]
	*Thalassiosira pseudonana*	[4,7]
	*Thalassiosira oceanica*	[7]
	*Thalassiosira rotula*	[7]
	*Thalassiosira aestivalis*	[7]
**Dinoflagellates**	*Alexandrium minnutum* ^b^	[6]
	*Gymnodinium catenatum* ^b^	[7]
	*Gymnodinium nagasakiense* ^b^	[9]
	*Peridinium cinctum* fa. *Westii*	[10]
	*Pyrodinium bahamense* ^b^	[11]
**Prymnesiophytes**	*Chrysochromulina breviturrita*	[5]
	*Chrysochromulina kappa*	[12]
	*Chrysochromulina brevefilum*	[12]
	*Chrysochromulina strobilus*	[12]
	*Chrysochromulina polylepis*^b^	[14,15]
	*Helladosphaera* sp.	[1]
	*Emiliania huxleyi*	[1]
	*Gephyrocapsa oceanica*	[1]
**Raphidophytes**	*Chattonella verruculosa* ^b^	[8]
**Chlorophytes**	*Platymonas subcordiformis*	[13]
**Chrysophytes**	*Aureococcus anophagefferens* ^b^	[16]

^a^ Modified from Doblin *et al.* [5] ^b^ Harmful algae.

## 2. Se Uptake Mechanism

In land plants, selenate is absorbed through the mediation of a sulfate transporter as a counterpart to sulfate transport [18]. On the other hand, there is only a little information on a selenite transporter in wheat and the green alga *C. reinhardtii*, that showed no requirement for Se [19,20]. Thus, to understand the higher growth-stimulating effect of selenite than selenate in *E. huxleyi* and how selenite is absorbed by cells, it is important to elucidate why marine microalgae such as *E. huxleyi* utilize the selenite ion.

**Figure 1:**
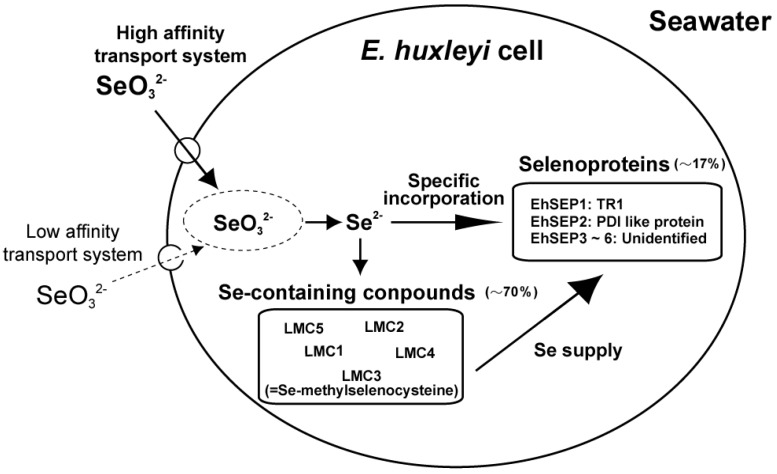
Selenium absorption and metabolic flow in *Emiliania huxleyi.* LMC1, 2, 4, and 5, unidentified Se-containing compounds; LMC3, Se-methyl-selenocysteine; EhSEP1, homologous to thioredoxin reductase 1; EhSEP2, homologous to protein-disulfide isomerase; EhSEP3-6, unidentified selenoproteins.

We investigated the uptake mechanism of selenite through kinetic analysis of ^75^Se-selenite uptake by *E. huxleyi* cells using a ^75^Se radiotracer technique. We found that two mechanisms are involved, namely an ATP-dependent active transport process with a high-affinity for selenite and a passive transport process with a low affinity for selenite [21]. The *K*_m_ of the active transport process for selenite was 29.8 nM. This *K*_m_ value suggests that selenite is taken up by cells via the active transport process at the surface of the ocean, where 0.1–0.2 nM selenite is present. In addition, selenite transport activity was not inhibited by selenate, sulfate, or sulfite ions, suggesting that selenite is transported using a potent transport mechanism (data not shown). Finally, *E. huxleyi* can efficiently absorb nanomolar levels of selenite via an ATP-dependent active transport process; this is why selenite has a higher growth-stimulating effect on *Emiliania* growth than selenate (Figure 1).

## 3. Metabolism of Se

**Figure 2:**
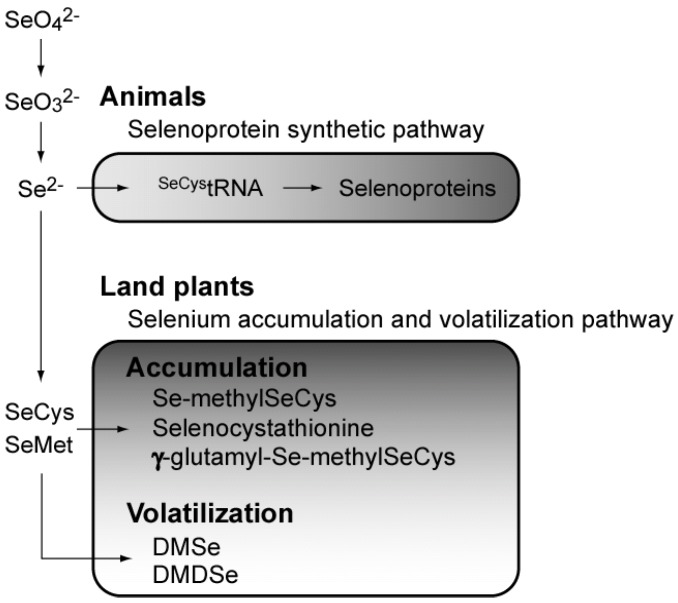
Overview of selenium metabolism. SeCys, selenocysteine; SeMet, selenomethionine; DMDSe, dimethyl-diselenide; DMSe, dimethylselenide.

Although Se is an essential element for many organisms, it is also toxic at higher concentrations. In Se-requiring organisms such as mammals, Se is utilized for the synthesis of abundant selenoproteins (Figure 2), but selenoproteins are not found in yeasts or land plants (Table 2).

**Table 2:** Distribution of selenoproteins in eukaryotes. ^a^

Phylum, Division	Species	Number of selenoproteins
**Nematoda**	*Caenorhabditis elegans*	1
	*Caenorhabditis briggsae*	1
**Arthropoda**	*Apis mellifera*	1
	*Drosophila melanogaster*	3
	*Drosophila pseudoobscura*	3
	*Anopheles gambiae*	3
**Chordata**	*Mus musclulus*	24
	*Homo sapiens*	25
	*Gallus gallus*	24
	*Xenopus tropicalis*	24
**Ascomycota**	*Schizosaccharomyces pombe*	0
	*Yarrowia lipolytica*	0
	*Saccharomyces cerevisiae*	0
**Dictyosteliomycota**	*Dictyosteliumd iscoideum*	5
**Anthophyta**	*Oryza sativa*	0
	*Medicago truncatula*	0
	*Populus trichocarpa*	0
	*Arabidopsis thaliana*	0
**Chlorophyta**	*Chlamydomonas reinhardtii*	12
	*Ostreococcus tauri*	26
	*Ostreococcus lucimarinus*	29
**Rhodophyta**	*Cyanidioschyzon merolae*	0
**Heterokontophyta**	*Thalassiosira pseudonana*	16
**Apicomplexa**	*Cryptosporidium parvum*	0
	*Plasmodium falciparum*	4
	*Plasmodium chabaudi*	4
	*Plasmodium yoelii*	4

^a^ Modified from Lobanov *et al.*, [34]

A few land plants have been found to accumulate Se up to a thousand-fold in the plant body [22], leading to the elucidation of the metabolic pathway of selenium, including Se accumulation and volatilization [23] (Figure 2). Land plants can metabolize inorganic Se ions to non-toxic organic compounds such as Se-methylselenocysteine and γ-glutamyl-Se-methylseleno-cysteine to reduce the toxic effects of Se [23].

However, there is no information on Se metabolism and very little information on selenoproteins in aquatic plants and algae. Using bio-positron-induced X-ray emission (bio-PIXE) analysis, we found that *E. huxleyi* concentrated Se 1,500-fold from media containing 10 nM selenite [24]. Se is an essential element in *E. huxleyi* cells, and we identified Se compounds accumulated in the cells by using a radio-labeling technique with ^75^Se-selenite. ^75^Se-labeled metabolites were analyzed using thin-layer chromatography (TLC) for the development of metabolites and radio-luminography for the detection of radioactive compounds. Cell components were separated into fractions of low-molecular mass compounds, proteins, lipids, polysaccharides, and nucleic acids. After a 16 h-incubation with ^75^Se-selenite in the light, *E. huxleyi* cells incorporated ca. 70% of ^75^Se into low-molecular mass compounds (LMCs), and ca. 17% was in the protein fraction. In TLC analysis of LMCs, Se-methylselenocysteine, which is a known non-toxic intermediate of selenium metabolism, was found among five ^75^Se-labeled compounds. However, ^75^Se-labeled selenite, selenocysteine, and selenomethionine were not detected in this experiment, suggesting confirmed that these seleno-amino acids are rapidly metabolized into non-toxic intermediates in *E. huxleyi* [21] (Figure 1). This was by a study reporting that the non-specific incorporation of selenocysteine and selenomethionine into proteins is a major reason for Se toxicity in land plants [25]. Our ^75^Se pulse-chase labeling experiment clearly showed that ^75^Se in LMCs was transferred into the protein fraction [21]. As seleno-amino acids are not directly incorporated into protein molecules in selenoprotein synthesis, these results suggest the possibility that ^75^Se-labeled compounds may be first metabolized to ^75^Se-selenide (Se^2-^) and then incorporated into selenoproteins through *de novo* selenoprotein synthesis in *E. huxleyi*, as found in mammals [21]*.*

## 4. Selenoproteins

The essential nutritional function of Se is due to the action of selenoproteins containing Se in the form of selenocysteine (Sec or SeCys); such selenoproteins play essential roles in maintaining cell viability [26,27]. Most identified selenoproteins are oxidoreductases that require strong nucleophilicity of Se at the catalytic site for activity [28]. Sec is co-translationally incorporated into proteins at the site of the UGA codon, which is usually used as a stop codon, located on mRNA when the Sec insertion sequence (SECIS) is also located in the 3'-untranslated region (UTR) of selenoprotein mRNAs [29]. Generally, the specific Sec-tRNA synthesized by the binding of selenide into Ser-tRNA contributes to insert Sec into the synthesized protein, with the help of Sec-specific elongation factor (EFsec) and SECIS-binding protein 2 [29]. The real stop codon is encoded as UAA downstream of the UGA codon on the mRNA. Although selenoproteins are present in diverse organisms, including bacteria, archaea, and eukaryotes (Table 2), neither selenoprotein genes nor any of the components of the Sec insertion mechanism have been found in the genomes of the plant *Arabidopsis thaliana* or the yeast *Saccharomyces cerevisiae* [30].

We now focus on physiological, biochemical, and molecular aspects of Se utilization by the unicellular coccolithophorid *E. huxleyi*, based on our studies, and then present an overview of the strategy of Se utilization by microalgae. Little information is available on selenoproteins in microalgae; ten selenoproteins have been found in the green alga *Chlamydomonas reinhardtii*, one in the diatom *T. pseudonana*, and two in the haptophyte *E. huxleyi* [30,31,32,33]*.* Bioinformatic approaches have identified other selenoproteins in the green algae *Ostreococcus tauri* and *O. lucimarinus* and in other species [34]. Interestingly, Se is essential for growth of *T. pseudonana* and *E. huxleyi*, but not for *C. reinhardtii*. In *C. reinhardtii,* glutathione peroxidase is usually synthesized as a Cys-containing protein (cysteine protein); however, a Sec-containing glutathione peroxidase (selenoprotein) is synthesized *de novo* when selenite is added to the culture medium. Such a flexible response of *C. reinhardtii* to Se availability may give it a great advantage, although the reason for this unique character in *Chlamydomonas* is still unclear. Other green algae such as *Osterococcus* also have selenoproteins, but Se essentiality and requirement have not been studied in these organisms. No selenoprotein gene was found in the database for the unicellular red alga *Cyanidioschyzone merolae*, which is a primary symbiotic photosynthetic organism and considered to be the origin of eukaryotes. Higher plants and yeasts also have no selenoproteins; therefore, characterization and identification of selenoproteins in Se-requiring photosynthetic organisms will help to elucidate evolutionary changes in strategies of Se utilization.

**Figure 3:**
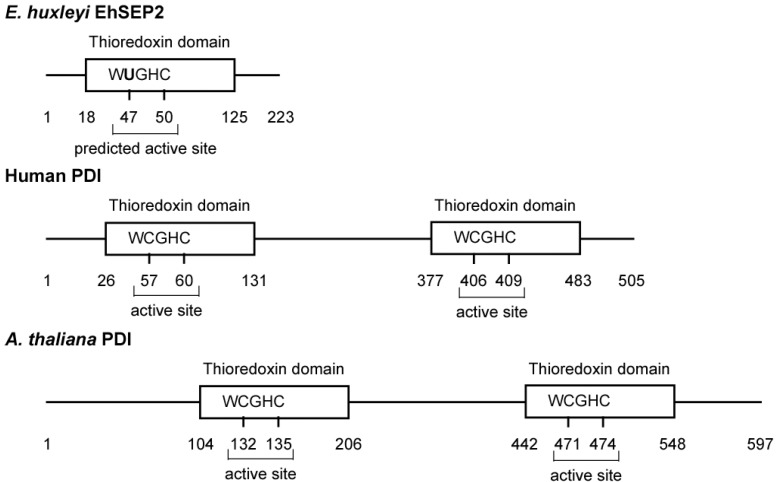
Scheme of some protein-disulfide isomerases. The PDIs and GenBank accession numbers of each sequence are BAD98262.1 (EhSEP2), NP_851234.1 (*Arabidopsis thaliana* PDI), and CAA89996.1 (*Homo sapiens* PDI).

In *E. huxleyi*, we found six selenoproteins (EhSEP1–EhSEP6) by using metabolic labeling techniques with ^75^Se. After incubating the cells with ^75^Se-selenite as substrate, ^75^Se-labeled proteins were detected by CBB staining and radioluminography on 2D-IEF/SDS-PAGE. First, the most abundant selenoprotein, EhSEP2, was identified as a protein-disulfide isomerase (PDI)-like protein [32]. PDI catalyzes the formation, reduction, and isomerization of protein disulfide bonds to regulate conformational changes and folding of the protein in the endoplasmic reticulum of eukaryotes. The PDI molecule possesses two well-conserved thioredoxin homology domains that contain two cysteine residues at the active site motif -CGHC- in mouse. However, *Emiliania* PDI-like protein (EhSEP2) contains only one thioredoxin homology domain, and Sec is located in a -UGHC- motif at a position corresponding to the active site motif of PDI (Figure 3).

**Figure 4:**
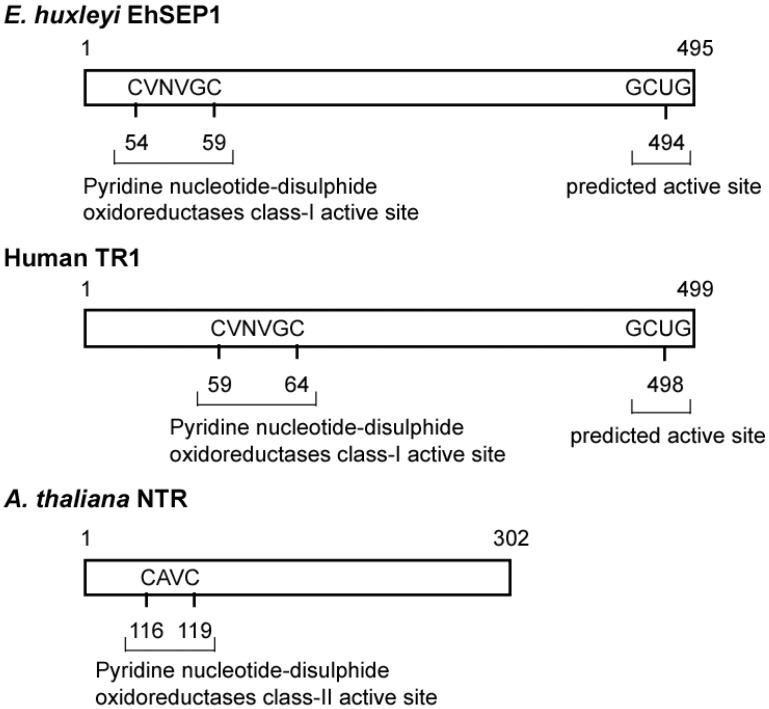
Scheme of some thioredoxin reductases. The TRs and GenBank accession numbers of each sequence are BAH20464.1 (EhSEP1), CAA80655.1 (*Arabidopsis thaliana* NTR), and AAH18122.2 (*Homo sapiens* TR1). Abbreviation: TRs, thioredoxin reductase.

Generally, the activity of PDI is dependent on the reactivity of cysteine residues in the active site motif located in the N-terminal region [35]. It is possible that EhSEP2 increases catalytic activity as a result of the replacement of cysteine by a Sec residue, because the strong nucleophilicity of Se makes it chemically more reactive than S. Thus, EhSEP2 is thought to maintain high reactivity even though it possesses only one thioredoxin homology domain. The second abundant selenoprotein, EhSEP1, was identified as thioredoxin reductase (TR) 1 [33]. TR1 is a very important enzyme because it reduces thioredoxin, which is known to adjust enzyme activity by oxidizing or reducing the disulfide bond in various enzymes (Figure 4). The induction of TR by Se was also reported in a green alga *Scenedesmus quadricauda* that is slightly resistant to Se-toxicity although no information of the involvement of Se in TR [36].

In mouse, deletion of TR1 leads to lethality [37]. The above evidence confirms results showing that TR1 is essential for growth of *E. huxleyi.* Thus, depending on the supply of Se, huge ocean blooms of this alga may be triggered by rapid growth and strong viability.

Eukaryotes have highly variable sets of selenoproteins, varying from zero in higher plants and fungi to more than 30 in some fish and algae. In addition, aquatic organisms tend to possess more selenoproteins than terrestrial organisms [34]. This may be because the utilization of selenium is easier in aquatic environments than terrestrial environments; thus, selenoproteins may have been lost during evolution from aquatic to terrestrial habitats. In particular, the loss of selenoproteins is marked in photosynthetic organisms. Therefore, more analysis of Se utilization in photosynthetic organisms, such as algae that require Se, offers a great opportunity to uncover evolutionary trends in selenium utilization and function by comparison with land plants or algae that do not require Se but possess selenoproteins.

## 5. Conclusions

Properties of Se requirement for growth and the Se-metabolism to selenoproteins and detoxified compounds are widely distributed among microalgae. The diversity in Se metabolism among various taxa may be due to their growth response and nutritional essentiality to Se. Land plants seem to lose Se-essentiality since Se distribution in land environment is greatly limited. The second symbiont alga, coccolithophorid *E*. *huxleyi* (Haptophycea) possesses properties of both animal-type selenoprotein synthesis and detoxified compound production via higher-plant-type metabolism. Such very unique properties are found mainly in *E. huxleyi* at this moment, but other second symbiont algae such as diatoms, dinoflagellates and other prymnesiophytes may also possess similar Se-utilization strategy. Marine microalga *E. huxleyi* absorbs selenite using an active transport system, even the concentration of selenite is lower than that of selenate in seawater. Selenite utilization will be advantageous for algae since selenite (IV) is a more reduced form of Se in comparison with selenate (VI). Such Se metabolism may be favorable for the growth of algae in Se-limited environment, as reported on iron utilization in diatoms [38].

PDI of *E. huxleyi* is a selenoprotein and possesses only one thioredoxin domain in which Se is located at the active site of the domain as selenocysteine. As PDI in other organisms is a sulfar-enzyme, not selenoprotein, this PDI is very unique. Such replacement of S to Se may increase in the enzyme activity since Se has stronger nucleophilicity to give higher reactivity to enzymes than S. Most selenoproteins found up to now are oxidoreductases that catalyze the reduction and oxidation of metabolites and proteins. Microalgae seem to obtain highly reactive enzymes as a result of the utilization of Se. TR1 is also selenoprotein in *E. huxleyi* and is also selenoprotein in other organisms. Some selenoproteins are same among species but species-specific variation also exists in each organism. It is important to understand the evolution of Se utilization in photosynthetic organisms that the search of selenoproteins especially in algae. Therefore, to elucidate why and how such species-specific strategy for Se-utilization has been introduced will be remained for further investigation.
